# Consumption of identically formulated foods extruded under low and high shear force reveals that microbiome redox ratios accompany canine immunoglobulin A production

**DOI:** 10.1111/jpn.13419

**Published:** 2020-07-23

**Authors:** Matthew I. Jackson, Christopher Waldy, Chun‐Yen Cochrane, Dennis E. Jewell

**Affiliations:** ^1^ Pet Nutrition Center Hill's Pet Nutrition Inc. Topeka KS USA; ^2^ Department of Grain Science and Industry Kansas State University Manhattan KS USA

**Keywords:** canine, immunoglobulin A, metabolome, microbiome, redox, resistant starch

## Abstract

Digestion‐resistant starch (RS) can provide health benefits to the host via gut microbiome‐mediated metabolism. This study tested the physiological effects on healthy dogs of identically formulated foods processed under high (*n* = 16) or low (*n* = 16) shear extrusion conditions resulting in respective lower and higher levels of RS. Faecal samples collected at weeks 3 and 6 were assayed for stool score, proximate analysis, short‐chain fatty acids (SCFA), immunoglobulin A (IgA) and microbiome; faecal metabolome was characterized at week 6. Proximate and digestibility analyses of the foods and stool scores and stool proximate analysis showed few differences between the two shear methods except for increased apparent fibre digestibility in the low shear food. In contrast, levels of butyrate (*p* = .030) and total SCFA (*p* = .043) were significantly greater in faeces at week 6 from dogs who consumed the low versus high shear food. Faecal IgA levels were significantly higher at week 3 (*p* = .001) but not week 6 (*p* = .110) in the low shear food. Significant differences in 166 metabolites between consumption of the two foods were identified via faecal metabolomic analysis, with changes in sugars, bile acids, advanced glycation end products and few amino acids. Strikingly, consumption of the low shear food resulted in elevated levels of the reduced members of redox couples derived from metabolized sugars and branched‐chain and phenyl amino acids. Alpha diversity of the microbiome showed significantly higher species richness in faeces from the low shear group at week 6, though other measures of diversity were similar for both foods. Twelve genus‐level operational taxonomic units (OTU; half Firmicutes) significantly differed between the food types. Six OTU significantly correlated with RS‐derived sugars and ratios of the redox couples. Taken together, these data show that RS impacts microbiome‐mediated metabolism in the gut, resulting in changes in the reducing state.

## 1 INTRODUCTION

Food processing can increase the digestibility and bioavailability of nutrients, but retaining digestion‐resistant starch (RS) can also provide health benefits. Extrusion is a method of processing mixed ingredients that aids in the production of a uniform food form capable of being delivered at commercial scale (Guy, 2003). The food is cooked via application of mechanical shear force and heat, both of which can be scaled on a spectrum from mild to intense during extrusion. Depending on the conditions utilized, extrusion can improve the digestibility of protein (Omosebi, Osundahunsi, & Fagbemi, 2018) and amino acids (Clarke & Wiseman, 2007) and increase fibre‐carbohydrate solubility by degrading plant cell walls (Ralet, Thibault, & Della Valle, 1991). At extremes of high intensity extrusion, the process can degrade amino acids, especially lysine, by the formation of irreversible advanced glycation end products (AGE) (Bjorck, Noguchi, Asp, Cheftel, & Dahlqvist, 1983) and can affect the absorption of minerals (Kivisto, Andersson, Cederblad, Sandberg, & Sandstrom, 1986) as well as the stability of vitamins (Riaz, Asif, & Ali, 2009).

Typically, decreased nutrient digestibility resulting from extrusion conditions is considered counterproductive from a nutritional perspective. However, mild conditions of grain extrusion with reduced mechanical shear force and heat result in retention of type 2 native RS, a form of starch in which the amylose molecules are tightly packed in a crystalline form that is resistant to mammalian digestive processes (Sajilata, Singhal, & Kulkarni, 2006). Additionally, mild extrusion can reduce the scission of high‐molecular‐weight starch polymers that are slower digesting than low molecular weight starch (Guha & Ali, 2002). As RS2, the external barrier of the grain is removed, yet intact granules are poorly digestible since the starch within is in a crystalline form that renders it inaccessible to digestive enzymes. By definition, RS is not hydrolysed in the upper gastrointestinal tract or small intestine (Sajilata et al., 2006) and thus arrives relatively intact to the colon/large intestine. Ingestion of RS has been shown to impact the physiology of animals, including humans (Maier et al., 2017) and dogs (Peixoto et al., 2018).

A diverse community of microbiota (i.e. the microbiome) resides within the colon of monogastric animals. In this mutualistic relationship, the host makes both nutrients and macromolecule effectors available to the microbiome. In addition to starch from undigested food, host cell surface‐bound saccharides can be utilized by the microbiome as a nutrient source (Hoskins, Boulding, Gerken, Harouny, & Kriaris, 1992; Pickard & Chervonsky, 2015). The host also generates and secretes soluble immunoglobin A (IgA), which can bind to selective microbiota, promote intestinal immune sampling, and maintain microbiome homoeostasis (Nakajima et al., 2018; Ruiz, Delgado, Ruas‐Madiedo, Margolles, & Sanchez, 2016).

The metabolic functions of the microbiome complement those of the host in both type and scale. Microbiome‐mediated metabolism can largely account for the differences between ileal and total‐tract (apparent) nutrient digestibility. In particular, the microbiome can carry out the saccharolysis of intact starch granules in the colon (Herrmann et al., 2018). The microbiome generates a number of small molecule metabolites, including short‐chain fatty acids (SCFAs) from fermentation (Koh, De Vadder, Kovatcheva‐Datchary, & Backhed, 2016; Morrison & Preston, 2016), that become available to the host and serve a number of functions (Aguilar‐Toaláa et al., 2018). In addition, the microbiome can carry out proteolysis and putrefaction of amino acids (Yao, Muir, & Gibson, 2016), convert host‐produced primary bile acids to secondary bile acids (Ridlon, Kang, Hylemon, & Bajaj, 2014), and metabolize plant phenols (Jackson & Jewell, 2019), all of which have a variety of physiological effects on the host.

In this study, the hypothesis was tested that consumption of foods produced with lower extrusion intensity (i.e. containing higher levels of RS) increases fermentative markers of the canine hindgut microbiome. To this end, we examined metabolomic markers relevant to gut microbiome processing of dietary nutrients including saccharolysis and fermentation of carbohydrates, proteolysis and putrefaction of protein, and bile acid conversion. Related physiological outcomes including microbiome community composition, digestibility and stool quality were also assessed.

## 2 MATERIALS AND METHODS

### 2.1 Food production

Identically formulated diets composed of corn, rice, egg, poultry meal, corn gluten and lard, which met the maintenance nutrition requirements outlined by the Association of American Feed Control Officials (AAFCO) and National Research Council, were extruded with either high or low shear force. Extrusion was performed at the highest or lowest maximal shear rates that would provide a finished kibble with appropriate aesthetic properties such as density, size and texture (friability) to ensure adequate acceptance of food by dogs. Elements of the extruder are outlined in Figure S1, which shows the pertinent parameters that differentiate low from high intensity extrusion: shaft speed, mass throughput, size of die open area and extrudate temperature. The outside diameter of the screw element for the low and high shear study was 115 mm, and the extruder was a Wenger model X‐115 with a Wenger model 7 preconditioner. Total energy (TE) in extrusion is the sum of both specific mechanical energy (SME) and specific thermal energy (STE), uncorrected for efficiency losses. The SME is calculated as the power a motor draws to extrude a mixture divided into the amount of mixture flowing through the extruder. For the high shear extrusion, the power and thermal inputs were 36 and 37 kW, respectively, each divided into a mixture flow rate of 887 kg/hr, leading to a calculated SME of 41 w.h/kg and a STE of 42 w.h/kg for a TE of 83 w.h/kg. In the low shear condition, power and thermal inputs were 16 and 75 kW, respectively, each divided into a mixture flow rate of 1826 kg/hr, leading to a calculated SME of 9 w.h/kg and a STE of 42 w.h/kg for a TE of 83 w.h/kg. This gave an approximately fourfold reduction in SME under the low shear parameters (Figure S1). The higher TE in the high shear extrusion was due to increased shaft speed, low mass throughput, reduced die open area and much higher extrudate temperature. After extrusion, the formulations were dried and coated with palatants.

### 2.2 Food analysis

Each food was initially screened for digestibility in separate digestibility studies following the AAFCO quantitative collection protocol (Association of American Feed Control Officials, 2019). In these trials, animals were fed to maintain body weight, and water was freely available to animals at all times. Each test included six adult dogs and consisted of two phases: a pre‐collection acclimation period of 5 days followed by a second phase lasting 5 days with total collection of faeces. Analytical measurements for food‐related energy, moisture, protein, fat, fibre, and ash and the corresponding nutrients in faeces were performed as outlined by AAFCO. Both foods provided acceptable stool quality and digestibility where a stool score of at least three out of five (scale described below) or greater is acceptable and digestibility of protein, fat and carbohydrate is >85%.

For rapid visco analysis (RVA), the samples were frozen at −70°C, ground and passed through a US#40 sieve (Crosbie & Ross, 2007). Moisture was measured and used to calculate sample size and water addition. Samples were run on a 23‐min profile and analysed as previously described (Martinez, 2015). Proximate analysis was carried out as previously (Jackson & Jewell, 2019).

### 2.3 Animals and experimental design

Healthy dogs, as monitored through clinical and physical indices, were included in this longitudinal study. All were housed at Ontario Nutrient Laboratories in Fergus, Ontario, Canada in pairs, were provided daily group exercise in outdoor grassy runs and had access to natural light. Exclusion criteria were recorded instances of gastrointestinal upset (vomiting and diarrhoea) or an abnormally low appetite. According to the protocol, any low food intake resulting in weight loss >15% required the dog to be removed from the study, or at the determination of the veterinarian on a case‐by‐case basis. All subjects completed the trial with no adverse events. The study protocol was reviewed and approved by the Institutional Animal Care and Use Committee, Hill's Pet Nutrition, Topeka, KS, USA (Protocol Number: FP592.1.1.0‐A‐C‐D‐ADH‐EXT‐66‐GI) and complied with the guides for the care and use of laboratory animals from the National Institutes of Health, US National Research Council and US Public Health Service.

Dogs were randomized by age, weight and sex into two groups, wherein one group received the high shear food and the other the low shear food and were fed to maintain body weight. Caretakers were blinded to the food received, and each dog received only one of the diets. Body weight was assessed at baseline and every week for the duration of the study.

The study was a randomized 6‐week longitudinal feeding trial. To better account for heterogeneity in faecal samples, two replicate faecal collections were carried out within a four‐day period for each dog during week 3 and week 6. Metabolomics analysis was carried out only on samples collected at week 6. Analytical values derived from replicate collections during weeks 3 or 6 were averaged for all endpoints prior to statistical analysis, except for permutational multivariate analysis of variance (PERMANOVA) and negative binomial mixed modelling of individual OTU count data, which used collection day in the models.

### 2.4 Faecal analysis

Stool scores were immediately assessed on a 5‐point scale on which a higher score indicates firmer stool, and values below three indicate subjectively poor stool quality (Hall, Melendez, & Jewell, 2013). After scoring, faeces were extensively homogenized and portions were flash frozen in liquid nitrogen within 30 min of defecation for SCFA, IgA, metabolomic and microbiome analysis. Faecal homogenate that was not flash frozen was analysed for moisture and ash. Organic dry matter was calculated as 100% subtracted from the sum of the moisture % and ash %. Faecal proximate analysis, SCFA analysis, metabolomics and the composition of the faecal microbiome were carried out as previously described (Jackson & Jewell, 2019). Metabolite values were median‐centred across samples by metabolite and presented for each sample as fold level relative to that median. Faecal IgA was assessed using the canine IgA enzyme‐linked immunosorbent assay quantitation set (Bethyl Laboratories, Montgomery, TX) in which frozen faecal samples were lyophilized then mixed with extraction buffer prior to clarifying by centrifugation. Assay procedures were performed on collected supernatants according to the manufacturer's recommendations.

### 2.5 Bioinformatics processing

The FASTQ files were obtained from a MiSeq instrument (Illumina, San Diego, CA, USA) in pairs. The files were pre‐processed into contigs, chimera removed, and bacterial taxonomic classification was obtained using Mothur software (Schloss et al., 2009) using a modified protocol of the MiSeq SOP (Kozich, Westcott, Baxter, Highlander, & Schloss, 2013) published on the Mothur website (MiSeq SOP, 2017) according to GreenGenes reference taxonomy (DeSantis et al., 2006). Bacterial OTUs were identified based on taxonomic hierarchy. To obtain the functional profile of the microbial community in the samples, OTU data were further processed using Phylogenetic Investigation of Communities by Reconstruction of Unobserved States (PICRUSt) (Langille et al., 2013) to produce 16S copy number‐corrected relative abundances, and metagenome functional predictions were mapped to Kyoto Encyclopedia of Genes and Genomes (KEGG) orthologs (Kanehisa & Goto, 2000). The following metabolic classes of functional orthologs were assessed in the microbiome communities of the samples after determination of relative abundances by grouping those KEGG orthologs present in the PICRUSt output into metabolism pathways based on the KEGG reference pathways for arginine, benzoate, butyrate, phenylalanine, propionate, tryptophan and tyrosine.

### 2.6 Statistical analysis

Independent samples *t* tests were performed at weeks 3 and 6 for faecal proximate analyses, SCFA and log‐transformed faecal IgA values and at week 6 for log‐transformed faecal metabolites (sugars, amino acids, bile acids, AGE and redox couples). Statistical *t* tests were performed in JMP Pro versions 12–13 to generate *p* values. To account for the high dimensionality of metabolomics data, resulting *p* values were used to generate false discovery rate (FDR)‐correcting *q* values using the “qvalue” function in the R package qvalue v2.14.1 for false discovery assessment (Storey, Bass, Dabney, & Robinson, 2014).

Global metabolome assessment was carried out on the Metaboanalyst platform v4.0 (Chong et al., 2018). Orthogonal partial least squares (OPLS) analysis was used to detect metabolome‐wide differences between low versus high shear‐fed dogs, and Random Forest was used to detect metabolite predictors of group identity (Random Forest number of trees = 2,000, number of predictors = 10, Randomness = On). To find metabolomic associations with RS catabolism, the Pattern Hunter component of Metaboanalyst 4.0 was used to generate Spearman rank correlations between the global set of detected metabolites and individual RS‐derived sugar metabolites or lactate.

The 16S copy number‐corrected OTU counts and PICRUSt‐predicted functional data were first filtered by prevalence. Only those OTUs that passed the 80% prevalence in at least one of the diet x week experimental groups were considered for statistical analysis. The counts of individual OTUs were analysed by negative binomial mixed models (Zhang et al., 2018) with fixed shear and time effects and random dog effect. Permutational multivariate analysis of variance (PERMANOVA) based on Manhattan distance was used to compare relative abundances of microbial compositions and pathway functional compositions between shear types and by time using the adonis function in the R vegan package (Oksanen et al., 2018) with 4,999 permutations. All p‐values were FDR adjusted according to the Benjamini and Hochberg procedure (Benjamini & Hochberg, 1995).

Alpha diversity indices were calculated on collection day averaged genus‐level count data in the R programming environment (R Core Team, 2018) using the R vegan package (Oksanen et al., 2018). Alpha diversity is presented as Hill numbers (Hill, 1973) of order q, which correspond to taxa richness (S; *q* = 0), exponential of the Shannon index (expShannon; *q* = 1) and the inverse of the Simpson index (invSimpson; *q* = 2). Pielou's Evenness (J) is also reported to provide insight into the relative dispersity of taxa abundance independent of the number of taxa detected. Together, these indices provide insight into microbiome community structure when taxa abundance is not considered in the diversity calculation (S), taxa contribute to the index according to their abundance (expShannon), and highly abundant taxa contribute to a greater degree to the diversity value (invSimpson).

To facilitate analysis of the correlation between week 6 genus‐level OTU and RS‐related sugars or redox couple ratios present in the week 6 metabolomics data set, collection day averaged genus‐level count data were centred natural log‐ratio (CLR) transformed (Aitchison, 1994) using the aldex.clr function in the R package ALDEx2 (Fernandes et al., 2014; Gloor et al., 2017). Prior to CLR transformation, zero counts were inflated using Bayesian‐multiplicative treatment (Martin‐Fernandez, Hron, Templ, Filzmoser, & Palarea‐Albaladejo, 2015) using the CZM attribute of the cmultRepl function in the R package zCompositions (Palarea‐Albaladejo & Martín‐Fernández, 2015, 2018). Subsequent to CLR transformation, correlations between all OTU found at week 6 and the four RS‐derived sugars (maltotetrose, maltotriose, maltose and glucose) present in the week 6 metabolomics data set were carried out in the Response Screening Platform with Cauchy robust fit in JMP software package (JMP®, version 14.2. SAS Institute Inc., Cary, NC). Pearson correlation adjusted *R* ^2^ as well as the mean, *SD* and *p* value of regression parameter estimates are reported for genus‐level OTU that were significantly correlated with at least three of four RS sugars (FDR‐corrected *p* value of <.05) and were known a priori to be associated with dietary RS based on existing literature.

## 3 RESULTS

### 3.1 Food production and analysis

Identically formulated diets composed of corn, rice, egg, poultry meal, corn gluten and lard (Table 1) were produced with either high or low shear extrusion force (Figure S1). During RVA, three peaks are observed that inform on the levels of (a) cooked/gelatinized starch, (b) uncooked starch (a mixture of RS) and (c) high‐molecular‐weight starch; the latter two forms of starch are known to be less digestible. The differences in these peaks for the high versus low shear foods indicated that greater levels of RS were present in the low shear food (Figure S2). In the first six minutes of the RVA curves, early peaks representing cooked starch were observed under both high and low shear conditions, but the area under the curve (AUC) was about one‐third less with the low shear food. At about 9.5 min in the RVA, a peak representing uncooked starch (RS) was seen in the low shear food. The final part of the RVA curve showed greater levels of high‐molecular‐weight starch in the low shear food, with a relative AUC almost twice that of the high shear food.

**TABLE 1:** Study food composition, proximate analysis and digestibility

	Extrusion condition	
	High shear	Low shear
Food composition (%)		
Corn, yellow, whole	41.72	
Rice, brewers	13.76	
Egg, dried pelleted	13.76	
Poultry by‐product meal	10.27	
Corn gluten	7.87	
Pork fat	3.04	
Cellulose	1.90	
Lactic acid	1.50	
Dicalcium phosphate	1.36	
Chicken liver digest	1.10	
Sodium chloride, iodized	0.61	
Potassium citrate	0.60	
Choline chloride (70%)	0.55	
Calcium carbonate	0.50	
Potassium chloride	0.50	
Beet pulp	0.41	
Soybean oil	0.30	
Vitamin mix	0.12	
Taurine	0.06	
Mineral mix	0.05	
Natural antioxidant	0.01	
Proximate analysis		
Moisture, %	7.6	7.5
Fat, %	12.2	13.4
Protein, %	23.4	23.4
Neutral detergent fibre, %	7.4	7.2
Ash, %	5.8	5.8
Crude fibre, %	2.7	2.6
Kcal/lb	2,177	2,054
Digestibility^a^		
Apparent dry matter digestibility, %	85.9 ± 0.3	85.2 ± 0.7
Apparent protein digestibility, %^b^	88.4 ± 0.3	86.6 ± 0.5
Apparent fat digestibility, %	92.2 ± 0.3	93.3 ± 0.5
Apparent fibre digestibility, %	20.1 ± 2.6	28.0 ± 4.2
Apparent carbohydrate digestibility, %	91.6 ± 0.1	90.3 ± 0.5
Apparent energy digestibility, %	88.7 ± 0.2	88.0 ± 0.7
Diet gross energy, kcal/kg	4,795	4,523
Diet metabolizable energy‐AAFCO tested, kcal/kg^b^	3,995 ± 25	3,726 ± 70
Nitrogen‐free extract calories, %	48.3	46.2
Protein calories, %	23.9	23.2
Fat calories, %	27.8	30.6

Abbreviation: AAFCO, Association of American Feed Control Officials.

^a^ Apparent digestibilities and metabolizable energy presented as mean ± standard error from digestibility trials (*n* = 6).

^b^ Values in row are significantly different by independent *t* test at *p* < .05.

For proximate analyses, moisture, fat, protein, neutral detergent fibre, ash, crude fibre or gross energy were similar between the high and low shear foods (Table 1). In parallel, results from the in vivo digestibility trials showed that when assessed by independent *t* test, the apparent per cent digestibility of dry matter, fat, carbohydrate and gross energy were not significantly different between the high and low shear foods. In contrast, although small in magnitude, the differences between foods for apparent protein digestibility (*p* = .02) and metabolizable energy (*p* < .001) reached statistical significance (Table 1). There was a 40% increase in apparent fibre digestibility in the low shear compared with the high shear food but this did not reach significance, likely due to the level of variation observed.

### 3.2 Study animals, food intake and weight

Thirty‐two dogs were included in the study and split evenly between the high and low shear food groups, with a mean (*SD*) age of 6.4 (3.7) years (Table S1). Most (69%) were beagles, with the rest of mixed and/or toy breed.

A longitudinal analysis of food intakes over the entire study showed no difference between the high and low shear foods (mean [SE], 124.4 [3.9] and 136.0 [4.2] kcal/kg^0.75^, respectively; *p* = .368). There was no change in weight compared with baseline (mean [*SD*], 12.8 [2.3] kg) for either the high shear (*p* = .956) or low shear (*p* = .355) food over the course of the study.

### 3.3 Stool scores and proximate analyses

Similar stool scores were observed for the high shear and low shear foods at week 3 (mean [SE], 4.5 [0.1] vs. 4.2 [0.1], respectively; *p* = .116) or week 6 (4.2 [0.1] vs. 4.1 [0.1], respectively; *p* = .549). In stool proximate analyses, there was no difference in stool moisture, ash or organic dry matter at weeks 3 or 6 between dogs who consumed high shear or low shear food (Table S2).

### 3.4 Faecal SCFA and IgA

At week 6, the levels of the straight‐chain SCFA butyrate as well as total SCFA were significantly higher in faecal samples from dogs that consumed the low shear food (Table 2). No differences by food type were observed in the levels of branched‐chain SCFAs (BSCFAs) at weeks 3 or 6.

**TABLE 2:** Fatty acid analyses from faecal samples taken at weeks 3 and 6 from dogs fed high or low shear food

Volatile fatty acid	Week 3			Week 6		
	High shear	Low shear	*p*, high versus low	High shear	Low shear	*p*, high versus low
Short‐chain						
Acetate, propionate, and butyrate	120.1 ± 6.1	123.8 ± 7.7	.574	108.1 ± 7.6	128.9 ± 9.6	.043
Acetate	64.7 ± 2.9	62.5 ± 2.2	.552	58.9 ± 3	67.5 ± 4.4	.119
Propionate	39.2 ± 2	43.8 ± 3.9	.302	34.5 ± 3.5	43.2 ± 4	.113
Butyrate	16.2 ± 1.2	17.5 ± 1.5	.523	14.7 ± 1	18.2 ± 1.1	.03
Branched chain						
2‐methylpropionate, 2‐methylbutyrate and 3‐methylbutyrate	6.4 ± 0.6	6 ± 0.5	.528	5.6 ± 0.5	5.4 ± 0.4	.765
2‐methylpropionate	2.5 ± 0.2	2.4 ± 0.2	.638	2.2 ± 0.2	2.1 ± 0.2	.829
2‐methylbutyrate	1.4 ± 0.1	1.3 ± 0.1	.533	1.2 ± 0.1	1.2 ± 0.1	.762
3‐methylbutyrate	2.6 ± 0.2	2.3 ± 0.2	.443	2.2 ± 0.2	2.1 ± 0.2	.712

Note

Values are mean ± standard error in μmol/g of undried faeces.

Faecal IgA levels from dogs fed the low shear food were significantly higher at week 3 (mean ± *SD*, 4.1 ± 0.1 vs. 3.5 ± 0.1 log10[IgA [ng]/faecal mass [mg]] in the high shear food; *p* = .001) but not at week 6 (4.1 ± 0.1 vs. 3.8 ± 0.1 log10[IgA [ng]/faecal mass [mg]]; *p* = .114).

### 3.5 Faecal metabolomics

Metabolomic analysis performed on faecal samples taken at week 6 from dogs fed the high or low shear food identified 584 metabolites. Of these, 166 were significantly different (*p* ≤ .05, *q* ≤ 0.1) between the high and low shear foods by independent samples *t* test, with 84 increased and 82 decreased (Table S3). The OPLS analysis indicated the presence of significant metabolome‐wide differences between dogs fed high and low shear foods when considering the 584 detected metabolites (Figure S3; *p* < 5 × 10^–4^ for both Q2 and RY2 parameters of the OPLS permutation); there were no instances of a test statistic with a larger magnitude than that of the observed data across 2,000 permutations. Random Forest analysis provided discrimination between the high and low shear food‐fed dogs with an overall out of bounds class error of 16%, where 14/16 dogs were correctly assigned to the high shear group (class error 13%) and 13/16 dogs were correctly assigned to the low shear group (class error 19%) (Figure S4). The top 20 ranked Random Forest predictors included catabolites of RS metabolism by gut microbes, bile acids, amino acid metabolites and amino acid‐derived redox couples (Figure S4). Strikingly, tocopherols and tocotrienols were also prominently ranked as Random Forest predictors and higher in faeces from the high shear group.

Based on the significance of metabolites in the results of the *t* test or appearance as predictors in the Random Forest analysis, the following metabolite classes were chosen for further analysis: saccharolysis and fermentation of carbohydrates, proteolysis and putrefaction of proteins, and bile acid conversion. Additionally, given the propensity of extrusion to generate AGE, the levels of these products in the low and high shear food‐fed groups were examined.

Sixteen saccharolytic metabolites were detected in faecal samples from week 6. Sugars that were significantly increased with consumption of the low shear food compared to the high shear food were potentially derived from microbial saccharolysis of RS (glucose, maltotriose and maltotetrose) and saccharolysis of intestinal epithelial cell surface glycosylated proteins (diacetylchitobiose, galactonate, mannose, and N‐acetylglucoseaminylasparagine). No sugars were significantly increased in faeces from dogs fed the high shear food compared with the low shear food (Table 3).

**TABLE 3:** Metabolites of interest detected via metabolomic screen in faecal samples taken at week 6 from dogs fed high or low shear food

Metabolite classes of interest	Metabolite class members	High shear	Low shear	*p* value, high versus low	Q value, high versus low
Saccharolysis of carbohydrates	Resistant starch derivative				
	Glucose	0.73 ± 0.02	1.02 ± 0.09	.010	.035
	Maltose	0.75 ± 0.17	1.06 ± 0.23	.290	.253
	Maltotetraose	0.76 ± 0.06	1.37 ± 0.16	.002	.013
	Maltotriose	0.45 ± 0.09	0.86 ± 0.13	.010	.033
	Epithelial sugar				
	Diacetylchitobiose	1.05 ± 0.13	1.93 ± 0.25	.002	.012
	Fucose	1.00 ± 0.24	1.49 ± 0.30	.090	.131
	Galactonate	0.60 ± 0.10	0.88 ± 0.13	.050	.089
	Mannose	0.60 ± 0.06	1.43 ± 0.27	.010	.028
	N‐acetylglucosaminylasparagine	0.84 ± 0.17	2.61 ± 0.93	.010	.034
	Other sugar				
	Arabinose	0.90 ± 0.09	0.88 ± 0.13	.590	.365
	Arabonate/xylonate	1.08 ± 0.14	1.36 ± 0.23	.430	.312
	Erythronate	1.22 ± 0.19	1.02 ± 0.11	.570	.356
	Fructose	0.87 ± 0.05	0.94 ± 0.08	.620	.372
	Glucuronate	1.18 ± 0.34	1.37 ± 0.14	.070	.108
	Threonate	1.27 ± 0.18	1.16 ± 0.16	.600	.366
	Xylose	0.89 ± 0.14	0.76 ± 0.18	.160	.183
Proteolysis of protein	Amino acids				
	Alanine	0.99 ± 0.13	0.81 ± 0.07	.260	.234
	Arginine	2.90 ± 1.49	3.20 ± 1.13	.560	.356
	Asparagine	1.26 ± 0.35	0.87 ± 0.17	.590	.365
	Aspartate	1.67 ± 0.35	1.01 ± 0.14	.140	.169
	Cysteine	1.07 ± 0.08	1.02 ± 0.08	.540	.352
	Cystine	1.10 ± 0.46	0.39 ± 0.08	.540	.352
	Glutamate	1.37 ± 0.24	0.88 ± 0.10	.049	.086
	Glutamine	1.04 ± 0.18	0.83 ± 0.09	.560	.356
	Glycine	1.20 ± 0.21	0.83 ± 0.08	.130	.162
	Histidine	0.89 ± 0.14	1.06 ± 0.11	.180	.193
	Isoleucine	0.99 ± 0.14	0.75 ± 0.07	.240	.226
	Leucine	0.94 ± 0.12	0.80 ± 0.08	.480	.332
	Lysine	1.11 ± 0.16	0.84 ± 0.06	.150	.173
	Methionine	1.01 ± 0.15	0.81 ± 0.08	.430	.311
	Phenylalanine	0.93 ± 0.11	0.80 ± 0.08	.460	.328
	Proline	1.20 ± 0.16	0.75 ± 0.05	.002	.014
	Serine	1.05 ± 0.16	0.82 ± 0.09	.440	.317
	Threonine	1.06 ± 0.16	0.80 ± 0.07	.350	.283
	Tryptophan	0.73 ± 0.13	1.02 ± 0.13	.140	.167
	Tyrosine	0.89 ± 0.12	0.83 ± 0.08	.840	.441
	Valine	1.02 ± 0.16	0.78 ± 0.07	.330	.273
Bile acid conversion	Primary bile acid				
	Cholate	1.28 ± 0.50	2.64 ± 0.43	.010	.027
	Cholate sulphate	1.01 ± 0.22	1.02 ± 0.20	.700	.397
	Glycochenodeoxycholate	0.45 ± 0.19	0.31 ± 0.09	.510	.341
	Glycocholate	3.21 ± 1.45	1.19 ± 0.23	.110	.144
	Tauro‐beta‐muricholate	8.54 ± 7.47	0.48 ± 0.10	.020	.050
	Taurochenodeoxycholate	1.17 ± 0.23	1.7 ± 0.37	.330	.274
	Taurocholate	5.85 ± 3.73	1.25 ± 0.24	.020	.048
	Secondary bile acid				
	12‐dehydrocholate	0.83 ± 0.24	3.28 ± 0.79	.001	.009
	3b‐hydroxy−5‐cholenoic acid	1.20 ± 0.14	0.94 ± 0.11	.130	.162
	3‐dehydrocholate	0.95 ± 0.41	1.82 ± 0.46	.080	.116
	6‐oxolithocholate	2.04 ± 0.32	1.53 ± 0.61	.020	.048
	7,12‐diketolithocholate	0.79 ± 0.13	1.74 ± 0.43	.030	.070
	7‐ketodeoxycholate	0.94 ± 0.32	3.95 ± 0.74	<.001	.003
	7‐ketolithocholate	0.59 ± 0.23	2.42 ± 0.32	<.001	<.001
	Dehydrolithocholate	1.54 ± 0.19	1.17 ± 0.31	.060	.094
	Deoxycholate	1.07 ± 0.06	0.97 ± 0.10	.240	.223
	Glycodeoxycholate	1.56 ± 0.23	0.75 ± 0.09	.001	.007
	Glycolithocholate	1.00 ± 0.15	0.43 ± 0.08	.001	.011
	Hyocholate	1.18 ± 0.21	1.52 ± 0.26	.130	.162
	Hyodeoxycholate	0.97 ± 0.09	1.26 ± 0.15	.540	.352
	Lithocholate	1.13 ± 0.05	0.92 ± 0.08	.100	.134
	Taurodeoxycholate	9.48 ± 6.49	1.22 ± 0.30	.010	.037
	Taurohyodeoxycholic acid	7.09 ± 4.28	1.18 ± 0.33	.040	.076
	Taurolithocholate	16.89 ± 14.8	0.99 ± 0.15	.030	.069
	Tauroursodeoxycholate	9.21 ± 6.04	1.1 ± 0.28	.070	.109
Extrusion processing of proteins	Advanced glycation end products or precursors				
	Lysine	1.11 ± 0.16	0.84 ± 0.06	.150	.173
	N6‐carboxyethyllysine	1.33 ± 0.09	1.03 ± 0.05	.010	.040
	N6‐carboxymethyllysine	1.44 ± 0.11	0.98 ± 0.09	.002	.015
	Pyrraline	0.75 ± 0.05	0.62 ± 0.03	.090	.128

Note

Values are mean (standard error) of relative fold levels based on median centring across samples for a given metabolite.

The levels of only two amino acids (glutamate and proline) were significantly increased in faeces from dogs fed the high versus low shear food (Table 3), and none were increased in the low versus high shear food, indicating that microbial proteolysis was largely not affected by shear.

Of the seven primary bile acids detected in the faecal metabolomics assay, one (cholate) was significantly increased with consumption of the low shear food and two (tauro‐beta‐muricholate and taurocholate) were significantly increased with the high shear food (Table 3). Eighteen secondary bile acids were detected, with four significantly increased in dogs fed the low shear food and six significantly increased with the high shear food.

Two lysine‐derived AGE (N6‐carboxyethyllysine and N6‐carboxymethyllysine) were significantly higher in faeces from dogs fed the high shear versus the low shear food (Table 3).

Nearly all observed metabolites that could be classified as microbial fermentative hydroxyl/keto redox couples (which would be expected to be in equilibrium with NADH/NAD^+^) were changed by shear condition (Table 4), and all ratios were altered. That is, consumption of the low shear food consistently produced elevated levels of the reduced (hydroxyl) member of the redox couple and lower levels of the oxidized (keto) member of the redox couple. This was observed for redox couples derived from the microbiome‐mediated metabolism of sugars as well as the branched‐chain (valine, isoleucine and leucine) and phenyl (phenylalanine and tyrosine) amino acids. At least one member of the redox couple was observed for the precursors histidine, methionine and tryptophan, and the levels of those forms were consistent with the low shear food promoting higher levels of the reduced form and lower levels of the oxidized form.

**TABLE 4:** Redox‐coupled congeners detected via metabolomic screen in faecal samples taken at week 6 from dogs fed high or low shear food

Form	Metabolite	High shear	Low shear	*p* value, high versus low	*Q* value, high versus low
Reduced	Lactate	0.50 ± 0.05	10.40 ± 2.86	<.001	.001
Oxidized	Pyruvate	1.18 ± 0.10	1.05 ± 0.05	.510	.341
Ratio	Lactate/pyruvate	0.51 ± 0.12	9.05 ± 2.24	.001	NA
Precursor	Isoleucine	0.99 ± 0.14	0.75 ± 0.07	.240	.226
Reduced	2‐hydroxy−3‐methylvalerate	0.60 ± 0.07	2.16 ± 0.45	.010	.024
Oxidized	3‐methyl−2‐oxovalerate	1.94 ± 0.65	0.82 ± 0.13	.040	.071
Ratio	2‐hydroxy−3‐methylvalerate/3‐methyl−2‐oxovalerate	0.58 ± 0.13	3.53 ± 0.99	.003	NA
Precursor	Leucine	0.94 ± 0.12	0.80 ± 0.08	.480	.332
Reduced	Alpha‐hydroxyisocaproate	0.62 ± 0.09	3.00 ± 0.69	.002	.012
Oxidized	4‐methyl−2‐oxopentanoate	1.82 ± 0.56	0.84 ± 0.11	.040	.072
Ratio	Alpha‐hydroxyisocaproate/4‐methyl−2‐oxopentanoate	0.62 ± 0.17	4.36 ± 1.21	.001	NA
Precursor	Valine	1.02 ± 0.16	0.78 ± 0.07	.330	.273
Reduced	Alpha‐hydroxyisovalerate	0.58 ± 0.07	1.90 ± 0.29	.001	.009
Oxidized	3‐methyl−2‐oxobutyrate	1.89 ± 0.51	0.84 ± 0.19	.010	.027
Ratio	Alpha‐hydroxyisovalerate/3‐methyl−2‐oxobutyrate	0.59 ± 0.20	3.56 ± 0.67	.001	NA
Precursor	Phenylalanine	0.93 ± 0.11	0.80 ± 0.08	.460	.328
Reduced	Phenyllactate	0.64 ± 0.09	2.49 ± 0.54	<.001	.002
Oxidized	Phenylpyruvate	1.68 ± 0.40	0.82 ± 0.12	.020	.047
Ratio	Phenyllactate/phenylpyruvate	0.76 ± 0.32	3.74 ± 0.96	.001	NA
Precursor	tyrosine	0.89 ± 0.12	0.83 ± 0.08	.840	.441
Reduced	3‐(4‐hydroxyphenyl)lactate	0.49 ± 0.14	2.89 ± 0.65	<.001	<.001
Oxidized	4‐hydroxyphenylpyruvate	1.39 ± 0.31	0.86 ± 0.08	.090	.128
Ratio	3‐(4‐hydroxyphenyl)lactate/4‐hydroxyphenylpyruvate	0.66 ± 0.34	3.71 ± 0.80	.001	NA
Precursor	Histidine	0.89 ± 0.14	1.06 ± 0.11	.180	.193
Reduced	Imidazole lactate	1.34 ± 0.38	2.29 ± 0.47	.030	.058
Precursor	Methionine	1.01 ± 0.15	0.81 ± 0.08	.430	.311
Oxidized	4‐methylthio−2‐oxobutanoate	1.62 ± 0.53	0.57 ± 0.21	.004	.021
Precursor	Tryptophan	0.73 ± 0.13	1.02 ± 0.13	.140	.167
Reduced	Indolelactate	0.61 ± 0.22	4.03 ± 1.05	<.001	<.001

Note

Due to the large number of possible precursors to lactate and pyruvate, they are not listed.

Values are mean (standard error) of relative fold levels based on median centring across samples for a given metabolite.

NA, not applicable; ratios were tested post hoc to global metabolomics testing and thus not subject to p value distribution required for q value generation.

In assessing the metabolomic changes associated with the catabolism of RS, global metabolite pattern screening with Metabolanalyst Pattern Hunter produced correlations between all detected metabolites versus each RS‐derived sugar (maltotetraose, maltotriose, maltose and glucose) or lactate. An inner‐join consensus list of metabolites that were significantly correlated with all four RS‐derived sugars as well as lactate at an FDR‐adjusted *p* value of .1 was generated and examined for metabolite class trends for association (Table S4). Out of 584 detected metabolites, 74 were associated with all four sugars as well as lactate (50 positive associations and 24 negative associations). Of these 74 metabolites, 34 could be grouped into three classes that were almost exclusively correlated in either a positive or negative association: lipids (primarily sphingolipids), microbial metabolites of plant secondary compounds (primarily phenols), and two‐electron redox couples (primarily the reduced forms). Regarding these classes, lipids were predominantly negatively associated with RS‐derived sugars and lactate, microbial metabolites of plant phenols were exclusively positively associated with these sugars and lactate, and the reduced forms of the redox couples were positively associated while the only correlated oxidized form was negatively associated. While amino acid metabolites also formed a prominent class in association with RS sugars and lactate, these were equitably distributed across both positive and negative correlations.

### 3.6 Faecal microbiome

A total of 313 OTUs were identified at the genus level in the faecal samples. Of this number, 68 passed the data filtering criteria and were used in the PERMANOVA and negative binomial mixed model analysis. As alpha diversity assessments include consideration of the presence/absence of taxa, all 313 OTU were included in these analyses.

Alpha diversity showed that there were differences in the faecal microbiome of dogs consuming the low shear versus high shear foods (Table 5). Species richness (S) was significantly higher in the low shear group at week 6 and was numerically higher at week 3. There were no significant differences between food shear types for expH, invSimp or J, though all were numerically greater in the low shear group at both timepoints.

**TABLE 5:** Alpha diversity metrics from faecal samples from dogs fed high or low shear food. Data are presented as mean ± standard error

Diversity index	Week 3			Week 6		
	High shear	Low shear	*p* value, high versus low	High shear	Low shear	*p* value, high versus low
S	77.44 ± 2.21	85.19 ± 3.23	.104	79.56 ± 1.83	91.13 ± 2.32	.002
expShannon	6.59 ± 0.40	7.96 ± 0.51	.096	9.17 ± 0.45	10.35 ± 0.35	.102
invSimpson	3.75 ± 0.27	4.32 ± 0.33	.262	6.08 ± 0.30	6.69 ± 0.24	.207
J	0.42 ± 0.01	0.45 ± 0.01	.211	0.50 ± 0.01	0.52 ± 0.01	.304

Abbreviations: expShannon, exponential of the Shannon index; invSimpson, inverse of the Simpson index; J, Pielou's evenness; S, taxa richness.

Assessment by PERMANOVA showed that the microbial composition was significantly different between diets and among time phases (*p* = .0003 for both). There were significant functional composition differences between diets and time phases in all seven pathways for all collections between week 3 versus week 6, except for collection 1 of week 3 versus collection 2 of week 6 (*p* < .01). There were no differences between the within‐week collections 1 versus 2 at week 3 or week 6. At the component level, of the 68 OTUs analysed, 12 showed significant differences between foods: 10 OTUs had higher relative abundances and 2 OTUs had lower relative abundances in the low shear group than in the high shear group (*p* < .05, Table 6). Half of those that were significantly different between shear types were in the phylum Firmicutes.

**TABLE 6:** Significant diet effects in twelve OTUs. Values are the estimated log ratio of relative abundances between faeces from dogs fed the low shear and high shear food ± standard error. FDR, false discovery rate; OTU, operational taxonomic unit

OTU string	Ln (Low Shear/High Shear)	*p* value (FDR‐corrected)
4342860_k_Bacteria_ p_Firmicutes_ c_Bacilli_ o_Gemellales_ f_Gemellaceae_ g_Gemella	3.56 ± 0.89	.001
1126448_k_Bacteria_ p_Actinobacteria_ c_Actinobacteria_ o_Actinomycetales_ f_Actinomycetaceae_ g_Trueperella	3.25 ± 0.73	<.001
1003206_k_Bacteria_ p_Proteobacteria_ c_Alphaproteobacteria_ o_Sphingomonadales_ f_Sphingomonadaceae_ g_Sphingomonas	2.79 ± 0.75	.002
1024529_k_Bacteria_ p_Firmicutes_ c_Clostridia_ o_Clostridiales_ f_Clostridiaceae	2.64 ± 0.62	.001
4441081_k_Bacteria_ p_Actinobacteria_ c_Coriobacteriia_ o_Coriobacteriales_ f_Coriobacteriaceae	2.55 ± 0.55	<.001
527323_k_Bacteria_ p_Proteobacteria_ c_Gammaproteobacteria_ o_Enterobacteriales_ f_Enterobacteriaceae_ g_Yersinia	2.50 ± 0.48	<.001
102407_k_Bacteria_ p_Bacteroidetes_ c_Bacteroidia_ o_Bacteroidales_ f_Bacteroidaceae_ g_Bacteroides	2.39 ± 0.28	<.001
4319416_k_Bacteria_ p_Proteobacteria_ c_Alphaproteobacteria_ o_Rhizobiales_ f_Bartonellaceae_ g_Bartonella	2.02 ± 0.62	.006
1122547_k_Bacteria_ p_Firmicutes_ c_Bacilli_ o_Bacillales_ f_Bacillaceae	1.80 ± 0.52	.004
839684_k_Bacteria_ p_Firmicutes_ c_Clostridia_ o_Clostridiales_ f_Lachnospiraceae_unclassified	1.28 ± 0.41	.008
1010876_k_Bacteria_ p_Firmicutes_ c_Clostridia_ o_Clostridiales_ f_Ruminococcaceae_ g_Oscillospira	−0.63 ± 0.16	.001
167741_k_Bacteria_ p_Firmicutes_ c_Clostridia_ o_Clostridiales_ f_Lachnospiraceae_ g_Dorea	−0.70 ± 0.15	<.001

### 3.7 Correlations between individual microbial taxa and metabolites

In order to assess potential microbial drivers of RS metabolism, correlation‐based response screening was carried out between all detected OTUs and the four RS‐derived sugars detected in the metabolomic data set (Table 7). Five OTUs known to be associated with dietary RS levels passed an FDR‐corrected *p* value cutoff of .05 for correlation with all four RS‐derived sugars while one additional OTU passing the significance threshold was associated with three of the four RS‐derived sugars. Of these six OTUs, two were negatively correlated with the RS sugars while four were positively correlated with these sugars. We further assessed these six OTUs for correlations with the six detected redox ratio couples to determine the degree to which they may be associated with redox homoeostasis in the gut (Table 7). Four of the six OTUs were significantly correlated with all six redox ratios, one was correlated with 5/6 redox ratios, and one was correlated with 2/6 redox ratios. In every case, the direction of association with redox ratios was identical to the direction of association of that OTU with the RS‐derived sugars.

**TABLE 7:** OTU correlations with RS‐derived sugars and redox ratios. Estimates are presented as mean ± standard error. OTU, operational taxonomic unit; RS, resistant starch; SE, standard error

OTU	RS‐derived Sugar					Redox ratio					
		Glucose	Maltose	Maltotriose	Maltotetraose	2‐hydroxy−3‐methylvalerate/ 3‐methyl−2‐oxovalerate	3‐(4‐hydroxyphenyl) lactate/ 4‐hydroxyphenylpyruvate	alpha‐hydroxyisocaproate/ 4‐methyl‐2‐oxopentanoate	alpha‐hydroxyisovalerate/ 3‐methyl‐2‐oxobutyrate	Lactate/ pyruvate	Phenyllactate/phenylpyruvate
100567 k_Bacteria p_Firmicutes c_Clostridia o_Clostridiales f_Ruminococcaceae g_Ruminococcus	Estimate ± *SE*	−0.06 ± 0.01	−0.16 ± 0.04	−0.15 ± 0.03	−0.11 ± 0.02	−0.27 ± 0.05	−0.29 ± 0.06	−0.29 ± 0.06	−0.26 ± 0.06	−0.24 ± 0.08	−0.23 ± 0.05
	*p* value	<.001	<.001	<.001	<.001	<.001	<.001	<.001	<.001	.005	.001
	*R* ^2^ adjusted	0.39	0.31	0.39	0.46	0.46	0.41	0.43	0.34	0.20	0.30
102407 k_Bacteria p_Bacteroidetes c_Bacteroidia o_Bacteroidales f_Bacteroidaceae g_Bacteroides	Estimate ± *SE*	0.03 ± 0.01	0.04 ± 0.03	0.06 ± 0.03	0.06 ± 0.01	0.17 ± 0.04	0.18 ± 0.05	0.19 ± 0.05	0.18 ± 0.05	0.17 ± 0.06	0.16 ± 0.04
	*p* value	.011	.222	.046	.003	.001	.001	.001	.001	.010	.002
	*R* ^2^ adjusted	0.17	0.01	0.09	0.22	0.31	0.28	0.30	0.28	0.17	0.25
167741 k_Bacteria p_Firmicutes c_Clostridia o_Clostridiales f_Lachnospiraceae g_Dorea	Estimate ± *SE*	−0.08 ± 0.02	−0.29 ± 0.08	−0.25 ± 0.06	−0.16 ± 0.04	−0.49 ± 0.1	−0.6 ± 0.1	−0.53 ± 0.11	−0.48 ± 0.12	−0.51 ± 0.14	−0.57 ± 0.08
	*p* value	.005	.001	.001	.001	<.001	<.001	<.001	<.001	.002	<.001
	*R* ^2^ adjusted	0.2	0.27	0.3	0.28	0.40	0.48	0.38	0.31	0.26	0.57
839684 k_Bacteria p_Firmicutes c_Clostridia o_Clostridiales f_Lachnospiraceae unclassified unclassified	Estimate ± *SE*	0.04 ± 0.01	0.11 ± 0.03	0.11 ± 0.02	0.07 ± 0.02	0.14 ± 0.05	0.18 ± 0.05	0.17 ± 0.05	0.21 ± 0.05	0.26 ± 0.06	0.17 ± 0.05
	*p* value	.003	.004	<.001	.001	.012	.004	.006	.001	<.001	.002
	*R* ^2^ adjusted	0.23	0.22	0.34	0.30	0.16	0.22	0.20	0.30	0.37	0.24
1066621 k_Bacteria p_Bacteroidetes c_Bacteroidia o_Bacteroidales f_Prevotellaceae unclassified unclassified	Estimate ± *SE*	0.05 ± 0.01	0.19 ± 0.05	0.12 ± 0.04	0.07 ± 0.03	0.19 ± 0.07	0.16 ± 0.09	0.20 ± 0.08	0.14 ± 0.08	0.15 ± 0.10	0.14 ± 0.08
	*p* value	.003	.001	.007	.035	.015	.084	.022	.116	.151	.090
	*R* ^2^ adjusted	0.23	0.3	0.19	0.11	0.15	0.06	0.13	0.04	0.03	0.06
4410166 k_Bacteria p_Bacteroidetes c_Bacteroidia o_Bacteroidales f_Prevotellaceae g_Prevotella s_copri	Estimate ± *SE*	0.09 ± 0.03	0.35 ± 0.11	0.3 ± 0.08	0.17 ± 0.06	0.34 ± 0.16	0.54 ± 0.17	0.48 ± 0.17	0.5 ± 0.17	0.37 ± 0.21	0.49 ± 0.15
	*p* value	.024	.004	.002	.011	.050	.004	.012	.007	.094	.003
	*R* ^2^ adjusted	0.13	0.21	0.25	0.16	0.09	0.21	0.16	0.18	0.06	0.22

## 4 DISCUSSION

This study examined the effects of canine consumption of two identical food formulations extruded under different shear force conditions on canine hindgut microbiome saccharolysis and fermentation of carbohydrates, proteolysis and putrefaction of proteins, and bile acid conversion. An approximately fourfold lower shear force in the low shear condition than in the high shear condition but with similar thermal energy allowed the examination of the impact on canine microbiome metabolism of food extruded at different shear but with nominally different thermal input. Since the low shear extrusion produced food with less cooked starch and markedly more of the less digestible native and high‐molecular‐weight starch, it is expected that there was more RS in the low shear food.

Consumption of the two foods shared several similar characteristics. Total‐tract digestibility in canines between the high and low shear foods was not markedly different, there were few differences in the proximate analyses of the food, and there was no statistical difference in caloric intake by the dogs, indicating that both foods were adequately palatable. There was also no significant change in body weight across the study in dogs fed either low or high shear foods. Taken together with the similar caloric intake, this indicates that there were not metabolic differences in the response to the calorie types in these foods. Furthermore, the foods produced by low versus high shear conditions did not produce marked differences in observed stool firmness (stool score) or macro‐analytical makeup.

There were extensive changes to the saccharolytic and fermentative capacity of the canine microbiome upon feeding low shear food. Changes in SCFAs, major outputs of gut microbiome fermentation (Koh et al., 2016; Morrison & Preston, 2016) that can also be produced by metabolism of lactate generated by catabolism of starch (Crost et al., 2018; Duncan, Louis, & Flint, 2004), were observed in the faeces from dogs fed the two food types in this study. Here, changes to straight‐chain fermentative SCFAs were observed at 6 weeks but not 3 weeks, indicating that the gut microbiomes may require a lead‐in period to acclimatize to the RS before they were competent to produce higher levels of SCFA. The greatest effect on individual SCFAs was to increase faecal levels of butyrate rather than acetate or propionate, which is consistent with the known molar ratio of conversion of RS to butyrate relative to other fibre sources (Pryde, Duncan, Hold, Stewart, & Flint, 2002). Similar to these results, another study in which dogs were fed high or low RS food for 60 days found higher levels of lactate and SCFAs, including butyrate, in faeces from dogs fed the high RS food (Peixoto et al., 2018). No effect of consumption of foods having low versus high shear was found on faecal putrefactive BSCFAs, indicating a specific effect of RS on fermentative SCFAs. There was no change in faecal levels of proteolytic branched‐chain amino acid precursors to BSCFAs with shear type.

Increased fermentative capacity was accompanied by higher levels of IgA. An apparent benefit to gut immunity was seen through increased levels of IgA at week 3 in faeces from dogs fed the low shear food compared with the high shear food. Subsequently, levels of IgA appeared to increase in the high shear food such that there was only a residual trend towards difference between the two foods at week 6. Consistent with this, another study found that faecal IgA levels did not differ at day 60 between dogs fed foods with low or high levels of RS (Peixoto et al., 2018). Increased IgA accompanied by higher levels of SCFA with RS intake has also been observed in prior studies in dogs (Peixoto et al., 2018) and rats (Morita, Tanabe, Sugiyama, Kasaoka, & Kiriyama, 2004).

Consumption of foods at low or high shear resulted in a statistically significant shift in approximately 30% of the faecal metabolome at 6 weeks, producing significant differences between the groups by global metabolome analysis. Metabolite predictors of shear type foods were largely related to microbial saccharolysis and proteolysis, bile acid metabolism (including microbial‐derived secondary bile acids), and redox couples of fermentative and amino acid metabolism. Several metabolites in each class were significantly impacted by shear type. Specifically, consumption of the low shear food appeared to increase saccharolytic, but not putrefactive, microbiome processes. This finding is in line with prior reports of a decrease in protein proteolysis and putrefaction as a consequence of RS availability in the colon, as RS encourages the growth of saccharolytic microbes at the expense of putrefactive microbes (Maier et al., 2017; Walker et al., 2011).

The levels of several bile acids were significantly different in faeces from dogs fed the high or low shear foods. Indeed, the gut microbiome can be thought of as an additional endocrine organ due to its ability to modify host‐produced bile salts that then affect both host and microbiome physiology (Ridlon & Bajaj, 2015). The present study found that several secondary bile acids, particularly ones conjugated with taurine, were significantly increased in the faeces from dogs fed the high shear food compared with the low shear food. Interestingly, the sulphite moiety of taurine can provide gut bacteria with a terminal electron acceptor, thus encouraging their growth (Ridlon et al., 2014). Resistant starch may also alter the activity of the host farnesoid X receptor (FXR), which regulates bile acid synthesis and enterohepatic circulation, in opposite ways (Ridlon & Bajaj, 2015). Cholate, which was significantly higher in faeces from low shear food‐fed dogs, can activate FXR, while the FXR antagonist tauro‐beta‐muricholate was higher in faeces from the high shear food‐fed dogs.

Given the propensity of shear in extrusion to effect production of AGE (Takeuchi et al., 2015), the ability of AGE to produce negative health effects such as increased inflammation and oxidative stress (Uribarri et al., 2015), and the growing recognition that AGE can influence gut microbiome composition (Snelson & Coughlan, 2019), we assessed their presence as well as the AGE‐precursor lysine. This study found significantly lower levels of two types of AGE in faeces from dogs fed the low shear (i.e. higher RS) food compared with the high shear food. However, the health consequences for the host from the exposure of the gut microbiome to AGE are relatively unknown.

Related to microbial fermentation of saccharolytic metabolites, perhaps the most unexpected observation in this study was the consistent shift towards the reduced state for NADH/NAD^+^ coupled hydroxyl/keto pairs derived from both glycolytic and amino acid precursors. To our knowledge, this consistent shift in faecal redox state has not been previously reported in any context, though an increase in the glycolysis of glucose to pyruvate with consumption of RS has been observed (Maier et al., 2017). The ratio of reduced to oxidized (hydroxyl/keto) was significantly increased in faeces from dogs fed the low shear food in every instance that both couples were observed. We propose that the availability of ample electrons produced through microbiome‐mediated metabolism of RS drives this shift towards hydroxyl forms and away from keto forms. Further, it might be expected that microbiota residing in an environment harbouring ample reducing equivalents derived from RS and generating high ratios of lactate/pyruvate (along with accompanying high ratios of NADH/NAD^+^) would exhibit decreased ADP‐ribosylation as a post‐translational protein modification since NAD^+^ is the source of ADP‐ribose (Cohen & Chang, 2018). Thus, high levels of lactate and NADH shift the equilibrium away from ADP‐ribosylation and its potentially detrimental effects such as bacterial toxin activity (Cohen & Chang, 2018) or inflammation (Krukenberg, Kim, Tan, Maliga, & Mitchison, 2015) and towards more positive activities like wound repair (Ghani, Wagner, Becker, Hunt, & Hussain, 2004). Additionally, lactate/pyruvate and associated hydroxyl/keto redox couples may serve as two‐electron redox shuttles that enable communication and equilibration of NADH/NAD^+^ in the host colon environment with impact on colonocyte metabolism. Transport of deprotonated redox couples could occur through colonocyte monocarboxylate transporters (Ritzhaupt, Wood, Ellis, Hosie, & Shirazi‐Beechey, 1998) but may also potentially occur via simple chemical diffusion of the hydrophobic acid forms. Although alpha‐hydroxy carboxylic acids have a notably lower pKa than simple aliphatic carboxylic acids, colonic luminal contents are compensatorily more acidic than blood or intracellular fluid, potentially leading to “ion trapping” of deprotonated anionic base forms of the redox couples after diffusion of the acid form into colonocytes (Baumer, Bittermann, Kluver, & Escher, 2017).

Increased capacity for saccharolysis and fermentation was accompanied by changes in canine hindcut microbiome community composition; consumption of the low shear food increased the number of taxa detected (species richness). The data indicate that low abundance taxa contributed to the increase in diversity with the low shear food. This is consistent with previous observations that RS‐metabolizing commensal gut microbiota can be present at low abundance and act as keystone species to initiate cascades of substrate availability for other gut microbes through the phenomenon of cross‐feeding (Ze, Le Mougen, Duncan, Louis, & Flint, 2013). There were significant differences by shear type in the abundance structure (beta diversity) as well as the functional capacity of the gut microbiome community, which may indicate a high‐level change to the habitat of the canine microbiome upon consumption of dietary RS, as well as altered ability to catabolize dietary substrates and produce metabolites that impact host health.

Perhaps, driving the changes in microbiome community structure, individual microbes (*Oscillospira*, *Bacteroides*, *Dorea*, and an unclassified genus in the Lachnospiraceae family) are known from previous studies to be associated with dietary RS levels (Herrmann et al., 2018; Sun, Su, & Zhu, 2016; Umu et al., 2015; Ze, Duncan, Louis, & Flint, 2012). Notably, despite the increase in butyrate levels seen with the low shear food, there did not appear to be increases in known butyrate‐producing bacteria relative to the high shear food (Pryde et al., 2002). The relatively small degree of change at the individual OTU level seen in this study is likely not the exclusive driver of the community‐based changes seen with altered beta diversity of microbiome abundance and function. Many more OTU may have minimally changed but not reached significance when assessed at an individual OTU basis but positively contributing to overall alterations in community structure. Furthermore, although it is often assumed that increased microbial diversity in the gut is beneficial (largely due to observations that gut diversity is decreased in pathogenic situations such as *Clostridioides difficile* infection), this idea has not been proven (Reese & Dunn, 2018). According to the mathematical formulae of diversity calculations, overall diversity would necessarily decrease if a probiotic known to provide health benefits was consumed and subsequently increased in proportional abundance at the expense of colonic commensals. Rather, the collective metabolic capacity of the microbiome may be more important in determining the impact on host physiology than the particular identities or diversities of the microbes present (Heintz‐Buschart & Wilmes, 2018). In addition, RS has been shown to induce metabolic changes in germ‐free mice, indicating that RS can exert an effect independently of the microbiome (Bindels et al., 2017).

Providing insight into the microbial drivers of the saccharolytic and fermentative changes induced by the low shear food, six OTUs were found to be significantly correlated with three to four of four sugars derived from RS, and further, with nearly all of the six observed ratios of two‐electron redox couples. Two showed negative correlations with the RS‐derived sugars, indicating that they decreased with increased consumption of RS, and these two OTUs were also negatively associated with the redox couple ratios. These six OTUs may be drivers of RS catabolism and NADH‐coupled redox homoeostasis or are perhaps indicative of RS‐responsive microbes harboured in the canine gut microbiome. We further found that of 74 metabolites associated primarily in only one direction with all four RS sugars as well as lactate, 34 were from only three metabolite classes: lipids, microbial metabolites of plant phenols and the two‐electron redox couples. Additionally, amino acid metabolites were prominently present in the list of metabolites associated with RS sugars and lactate, but these were distributed across both positive and negative associations rather than being largely associated in one direction like the aforementioned three categories of metabolites. Other studies have correlated metabolites with members of the microbiome, finding associations of certain bacteria with lipids, sugars and/or amino acids (Maier et al., 2017; Sun et al., 2016; Umu et al., 2015). It may be that plant phenols, known to be bound to plant cell wall matrices, are liberated during the process of RS granule catabolism and made available to gut microbes for further metabolism (Jackson & Jewell, 2019).

In summary, the extrusion of grain‐containing canine foods at two different levels of shear force led to apparent increases in levels of uncooked/native starch in the low shear condition; it should be noted that a weakness of this study is that RS and starch were not measured directly, but rather were assessed indirectly by viscosity analysis. Consumption of the low shear food resulted in a multitude of effects in dogs that appeared to be driven by microbiome metabolism including saccharolysis and fermentation of carbohydrates, proteolysis and putrefaction of protein, host bile acid conversion, and redox conversion of amino acid metabolites. These microbiome metabolic features were accompanied by increased levels of IgA, which indicates that RS impacts host‐microbiome mutualism.

## ANIMAL WELFARE STATEMENT

The authors confirm that the ethical policies of the journal, as noted on the journal's author guidelines page, have been adhered to and the appropriate ethical review committee approval has been received. The authors confirm that they have followed standards for the protection of animals used for scientific purposes. The study protocol was reviewed and approved by the Institutional Animal Care and Use Committee, Hill's Pet Nutrition, Topeka, KS, USA (Protocol Number: FP592.1.1.0‐A‐C‐D‐ADH‐EXT‐66‐GI) and complied with the guides for the care and use of laboratory animals from the National Institutes of Health, US National Research Council, and US Public Health Service.

## Supporting information

Fig S1

Fig S2

Fig S3

Fig S4

Table S1

Table S2

Table S3

Table S4
