# Supplementary figures and images for: Consumption of identically formulated foods extruded under low and high shear force reveals that microbiome redox ratios accompany canine immunoglobulin A production
Source: J Anim Physiol Anim Nutr (Berl). 2020 Jul 23;104(5):1551–67. doi: 10.1111/jpn.13419 (PMC7540571; doi:10.1111/jpn.13419)

**(a)**

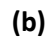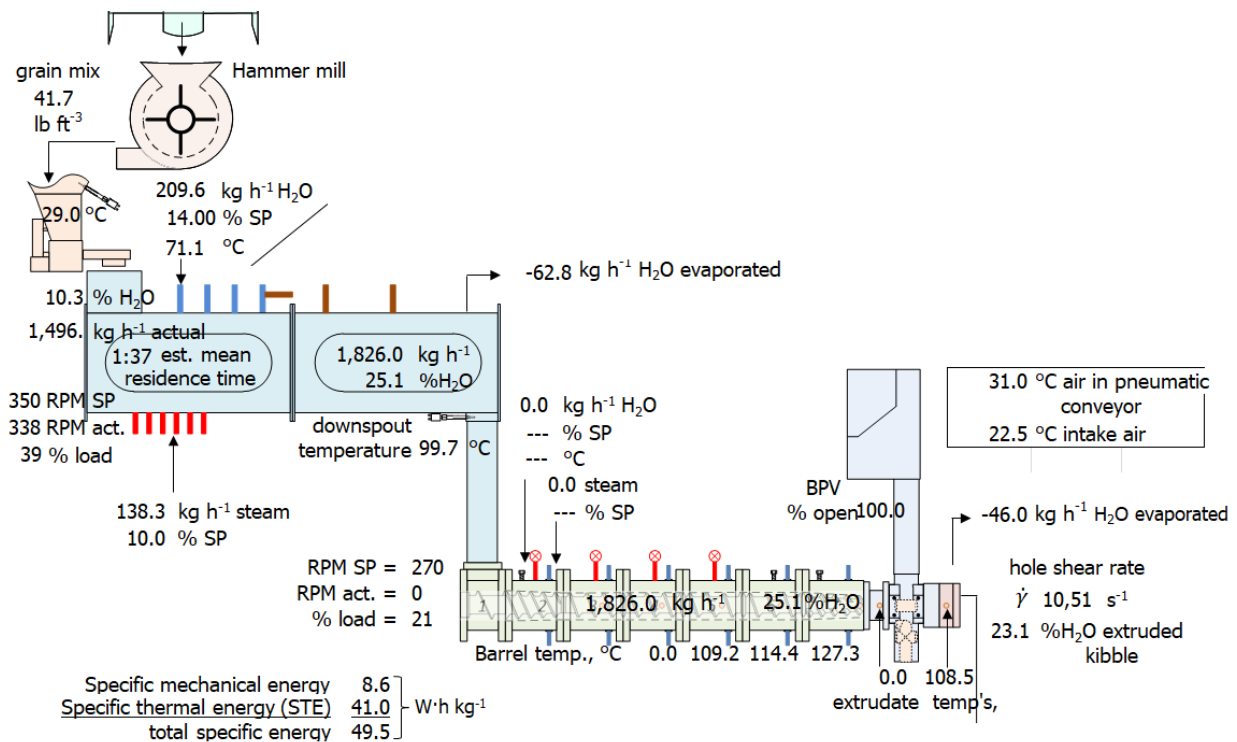

Supplement: Supplementary file 1 — Fig S1 [file JPN-104-1551-s001.pdf]
