# Supplementary material for: Consumption of identically formulated foods extruded under low and high shear force reveals that microbiome redox ratios accompany canine immunoglobulin A production
Source: J Anim Physiol Anim Nutr (Berl). 2020 Jul 23;104(5):1551–67. doi: 10.1111/jpn.13419 (PMC7540571; doi:10.1111/jpn.13419)
Supplement: Supplementary file 2 — Fig S2 [file JPN-104-1551-s002.pdf]

Supplementary Figure 2. Rapid visco analysis of the high shear and low shear foods over time. A, cooked starch; B, native starch; C, high MW starch. AUC, area under the curve; MW, molecular weight.

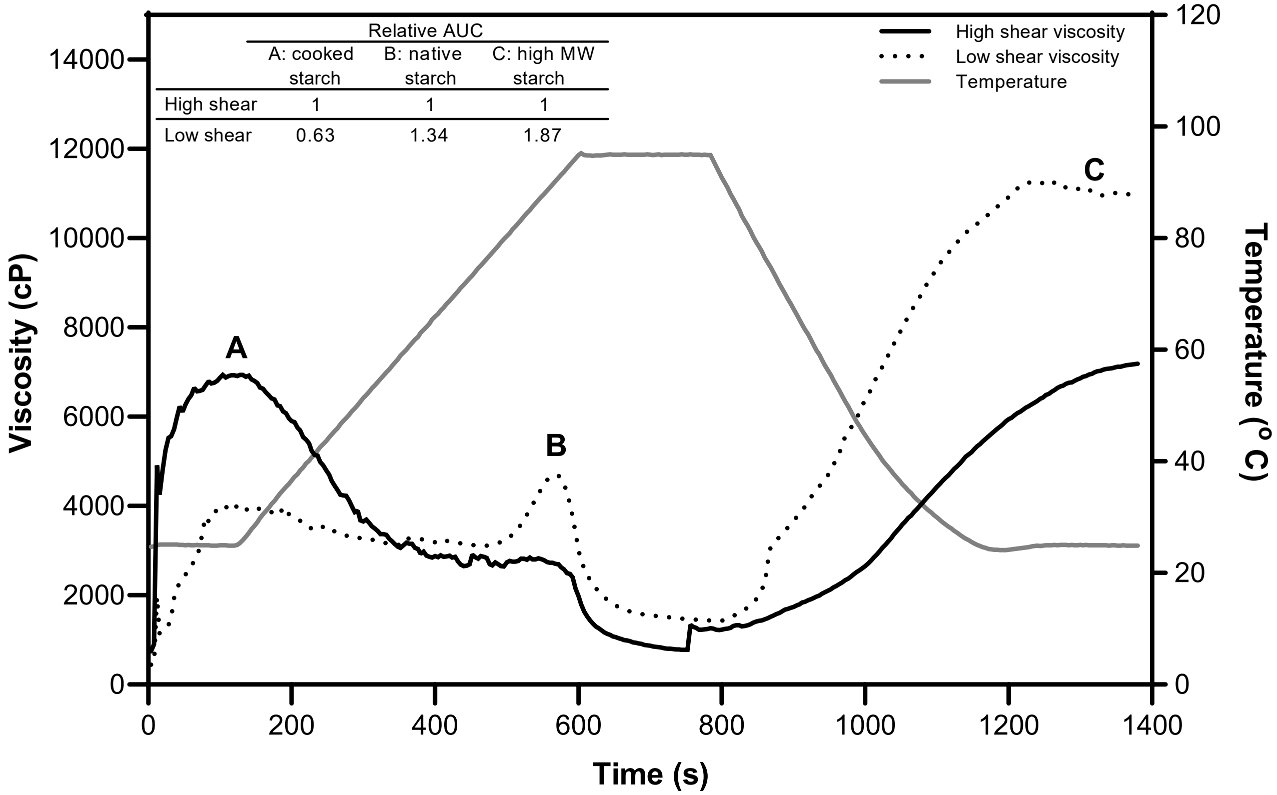

	Relative AUC		
	A: cooked starch	B: native starch	C: high MW starch
High shear	1	1	1
Low shear	0.63	1.34	1.87
