# Supplementary material for: Consumption of identically formulated foods extruded under low and high shear force reveals that microbiome redox ratios accompany canine immunoglobulin A production
Source: J Anim Physiol Anim Nutr (Berl). 2020 Jul 23;104(5):1551–67. doi: 10.1111/jpn.13419 (PMC7540571; doi:10.1111/jpn.13419)

Supplementary Figure 3. Orthogonal partial least squares (OPLS) analysis of whole fecal metabolome differences between low and high shear food-fed dogs. Shaded areas indicate the 95% confidence regions.

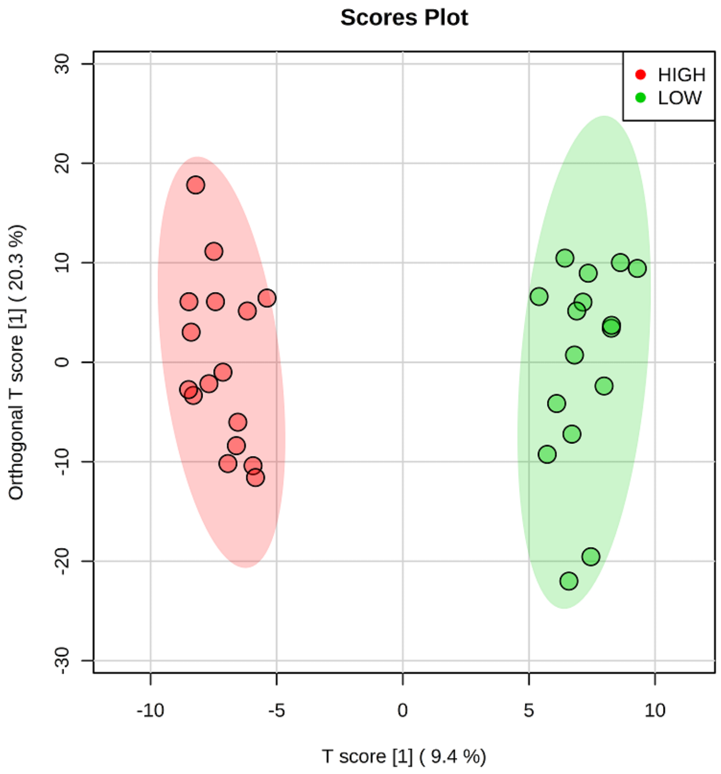

Supplement: Supplementary file 3 — Fig S3 [file JPN-104-1551-s003.pdf]
