# Supplementary material for: Consumption of identically formulated foods extruded under low and high shear force reveals that microbiome redox ratios accompany canine immunoglobulin A production
Source: J Anim Physiol Anim Nutr (Berl). 2020 Jul 23;104(5):1551–67. doi: 10.1111/jpn.13419 (PMC7540571; doi:10.1111/jpn.13419)
Supplement: Supplementary file 4 — Fig S4 [file JPN-104-1551-s004.pdf]

Supplementary Figure 4. Random Forest analysis of predictors of group differences. Red indicates the condition in which the metabolites are predicted to be higher; green is lower.

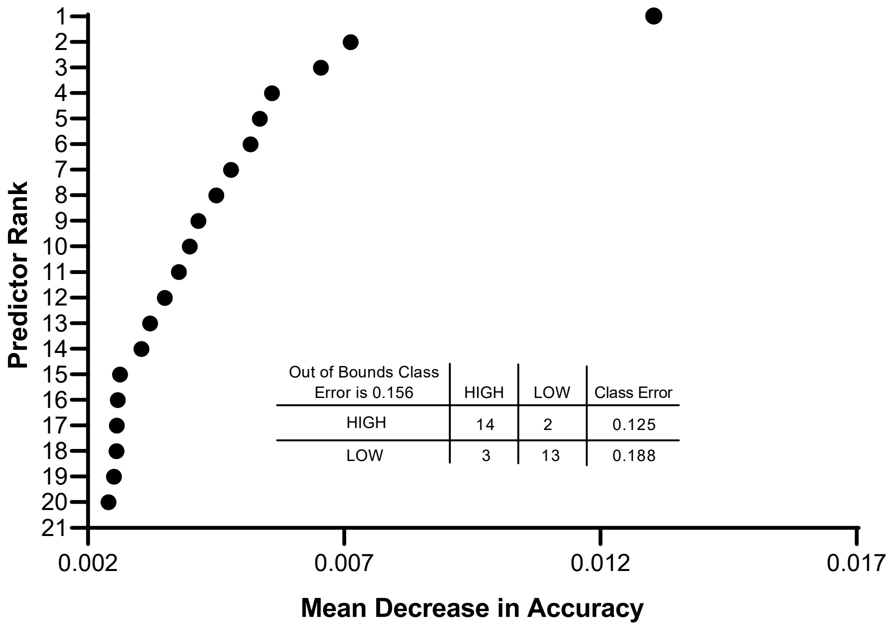

Random Forest Predictor	Predictor Rank	Mean Decrease Accuracy	High Shear	Low Shear
gamma-tocopherol/beta-tocopherol	1	0.013		
oleanolate	2	0.007		
alpha-hydroxyisovalerate	3	0.007		
alpha-tocotrienol	4	0.006		
glycodeoxycholate	5	0.005		
lactate	6	0.005		
1-palmitoleoyl-3-oleoyl-glycerol	7	0.005		
glucose	8	0.005		
2-hydroxybutyrate/2-hydroxyisobutyrate	9	0.004		
4-guanidinobutanoate	10	0.004		
alpha-hydroxyisocaproate	11	0.004		
indolelactate	12	0.004		
3-4-hydroxyphenyllactate HPLA	13	0.003		
3-methylhistidine	14	0.003		
7-ketolithocholate	15	0.003		
1-palmitoylglycerol 160	16	0.003		
N-acetylcysteine	17	0.003		
2-hydroxy-3-methylvalerate	18	0.003		
phenyllactate PLA	19	0.003		
gamma-tocotrienol	20	0.002		
