# Supplementary material for: Consumption of identically formulated foods extruded under low and high shear force reveals that microbiome redox ratios accompany canine immunoglobulin A production
Source: J Anim Physiol Anim Nutr (Berl). 2020 Jul 23;104(5):1551–67. doi: 10.1111/jpn.13419 (PMC7540571; doi:10.1111/jpn.13419)
Supplement: Supplementary file 5 — Table S1 [file JPN-104-1551-s005.pdf]

Supplementary Table 1. Baseline characteristics of canines in the study.

Subject number	Diet Type	Sex	Breed	Age (y)	Weight (kg)
1	High Shear	Male/neutered	Beagle	4.88	14.12
2	High Shear	Male/neutered	Mixed, Toy	6.71	14.27
3	High Shear	Male	Beagle	3.61	12.16
4	High Shear	Male	Beagle	3.60	12.76
5	High Shear	Female/spayed	Mixed	8.24	14.60
6	High Shear	Female	Beagle	3.56	10.90
7	High Shear	Male/neutered	Beagle	12.65	15.27
8	High Shear	Male/neutered	Beagle	3.57	13.64
9	High Shear	Female/spayed	Mixed	7.52	9.07
10	High Shear	Female/spayed	Mixed, Toy	4.04	11.15
11	High Shear	Male/neutered	Beagle	1.93	10.00
12	High Shear	Female/spayed	Mixed	7.24	12.85
13	High Shear	Male/neutered	Mixed	7.00	18.08
14	High Shear	Male/neutered	Beagle	3.59	11.55
15	High Shear	Female/spayed	Beagle	3.56	12.31
16	High Shear	Male/neutered	Beagle	3.60	10.27
17	Low Shear	Female/spayed	Beagle	3.56	10.27
18	Low Shear	Male/neutered	Beagle	3.55	9.68
19	Low Shear	Female/spayed	Mixed	8.43	15.56
20	Low Shear	Female/spayed	Mixed, Toy	4.10	14.69
21	Low Shear	Male/neutered	Beagle	11.75	14.72
22	Low Shear	Female	Beagle	3.58	9.67
23	Low Shear	Female	Mixed, Toy	4.04	14.24
24	Low Shear	Female/spayed	Beagle	13.29	11.74
25	Low Shear	Female/spayed	Mixed	8.39	14.62
26	Low Shear	Male/neutered	Beagle	3.62	10.17
27	Low Shear	Female/spayed	Beagle	12.39	12.22
28	Low Shear	Male/neutered	Beagle	13.46	15.44
29	Low Shear	Male/neutered	Beagle	8.45	17.12
30	Low Shear	Male	Beagle	3.36	12.06
31	Low Shear	Male/neutered	Beagle	14.10	12.24
32	Low Shear	Male/neutered	Beagle	3.62	10.60
