# Supplementary material for: Consumption of identically formulated foods extruded under low and high shear force reveals that microbiome redox ratios accompany canine immunoglobulin A production
Source: J Anim Physiol Anim Nutr (Berl). 2020 Jul 23;104(5):1551–67. doi: 10.1111/jpn.13419 (PMC7540571; doi:10.1111/jpn.13419)
Supplement: Supplementary file 6 — Table S2 [file JPN-104-1551-s006.pdf]

Supplementary Table 2. Stool proximate analyses at weeks 3 and 6 from dogs that consumed high or low shear foods.

	**Week 3**			**Week 6**		
	**High shear**	**Low shear**	**P value, high vs low**	**High shear**	**Low shear**	**P value, high vs low**
Ash	7.85 ± 0.22	7.69 ± 0.24	0.636	7.49 ± 0.24	7.87 ± 0.25	0.279
Moisture	67.32 ± 0.61	68.27 ± 0.55	0.254	68.40 ± 0.67	68.36 ± 0.55	0.966
Organic dry matter	24.83 ± 0.42	24.04 ± 0.38	0.167	24.11 ± 0.44	23.77 ± 0.33	0.540

Values are mean ± standard error.
