# Supplementary material for: Consumption of identically formulated foods extruded under low and high shear force reveals that microbiome redox ratios accompany canine immunoglobulin A production
Source: J Anim Physiol Anim Nutr (Berl). 2020 Jul 23;104(5):1551–67. doi: 10.1111/jpn.13419 (PMC7540571; doi:10.1111/jpn.13419)
Supplement: Supplementary file 8 — Table S4 [file JPN-104-1551-s008.pdf]

Supplementary Table 4. Significant Spearman rank correlations between resistant starch-derived sugars or lactate and metabolites. Blue shading indicates redox pairs; yellow, polyphenol metabolites; orange, lipids. FDR, false discovery rate; RS, resistant starch.

	Maltotetraose		Maltotriose		Maltose		Glucose		Lactate	
**Metabolite correlated with RS sugar or lactate**	**Spearman Rank Coefficient**	**FDR p value**	**Spearman Rank Coefficient**	**FDR p value**	**Spearman Rank Coefficient**	**FDR p value**	**Spearman Rank Coefficient**	**FDR p value**	**Spearman Rank Coefficient**	**FDR p value**
2-hydroxybutyrate/2-hydroxyisobutyrate	0.60	0.005	0.59	0.003	0.45	0.060	0.66	0.001	0.94	<0.001
alpha-hydroxyisovalerate	0.75	<0.001	0.68	<0.001	0.56	0.010	0.77	<0.001	0.92	<0.001
N-acetylcysteine	0.72	<0.001	0.66	0.001	0.43	0.080	0.63	0.002	0.90	<0.001
alpha-hydroxyisocaproate	0.72	<0.001	0.64	0.001	0.57	0.010	0.81	<0.001	0.89	<0.001
phenyllactate	0.68	0.001	0.68	<0.001	0.51	0.030	0.73	<0.001	0.89	<0.001
3-4-hydroxyphenyllactate	0.74	<0.001	0.71	<0.001	0.53	0.020	0.72	<0.001	0.88	<0.001
phenylalanylalanine	0.74	<0.001	0.76	<0.001	0.69	0.002	0.78	<0.001	0.85	<0.001
2-hydroxy-3-methylvalerate	0.65	0.002	0.56	0.010	0.56	0.010	0.73	<0.001	0.85	<0.001
indolelactate	0.67	0.002	0.75	<0.001	0.53	0.020	0.63	0.002	0.85	<0.001
valylleucine	0.63	0.003	0.65	0.001	0.64	0.003	0.82	<0.001	0.81	<0.001
leucylalanine	0.56	0.010	0.69	<0.001	0.63	0.005	0.71	<0.001	0.80	<0.001
pyroglutamine	0.50	0.020	0.72	<0.001	0.50	0.030	0.60	0.004	0.77	<0.001
N-acetylkynurenine 2	0.54	0.010	0.65	0.001	0.48	0.040	0.58	0.010	0.77	<0.001
phenylalanylglycine	0.63	0.003	0.71	<0.001	0.66	0.002	0.77	<0.001	0.76	<0.001
lysylleucine	0.72	0.001	0.70	<0.001	0.69	0.002	0.85	<0.001	0.75	<0.001
alanylleucine	0.65	0.002	0.67	0.001	0.71	0.001	0.84	<0.001	0.75	<0.001
leucylglutamine	0.61	0.005	0.69	<0.001	0.65	0.003	0.74	<0.001	0.75	<0.001
glycerophosphoethanolamine	0.56	0.010	0.67	0.001	0.51	0.030	0.64	0.002	0.73	<0.001
tryptophylglycine	0.56	0.010	0.68	<0.001	0.65	0.003	0.63	0.002	0.73	<0.001
hesperetin	0.63	0.003	0.65	0.001	0.48	0.040	0.62	0.003	0.73	<0.001
1-linoleoyl-GPC 182	0.68	0.001	0.71	<0.001	0.64	0.004	0.72	<0.001	0.72	<0.001

agmatine	0.65	0.002	0.72	<0.001	0.46	0.060	0.63	0.002	0.72	<0.001
N-acetylalanine	0.58	0.010	0.61	0.002	0.46	0.060	0.63	0.002	0.72	<0.001
equol sulfate	0.57	0.010	0.57	0.005	0.41	0.090	0.70	<0.001	0.70	<0.001
valylglutamine	0.50	0.020	0.61	0.002	0.43	0.080	0.68	0.001	0.70	<0.001
glutamine-leucine	0.53	0.020	0.60	0.003	0.62	0.010	0.67	0.001	0.70	<0.001
N-acetylarginine	0.63	0.003	0.74	<0.001	0.56	0.010	0.61	0.003	0.67	<0.001
leucylglycine	0.47	0.040	0.56	0.010	0.56	0.010	0.69	0.001	0.65	0.001
N6-formyllysine	0.49	0.020	0.66	0.001	0.59	0.010	0.62	0.002	0.65	0.001
thiamin	0.53	0.020	0.63	0.001	0.51	0.030	0.44	0.050	0.63	0.001
N-acetylglutamine	0.67	0.002	0.64	0.001	0.56	0.010	0.47	0.030	0.62	0.002
nicotinate ribonucleoside	0.58	0.010	0.60	0.002	0.47	0.050	0.70	<0.001	0.60	0.002
choline	0.64	0.003	0.60	0.002	0.45	0.060	0.52	0.010	0.60	0.002
4-hydroxycinnamate	0.64	0.003	0.58	0.003	0.47	0.050	0.56	0.010	0.58	0.003
threonylphenylalanine	0.54	0.010	0.58	0.004	0.73	<0.001	0.70	<0.001	0.57	0.004
histamine	0.58	0.010	0.70	<0.001	0.67	0.002	0.59	0.004	0.56	0.010
vanillate	0.51	0.020	0.47	0.020	0.41	0.090	0.62	0.002	0.54	0.010
1-methylhistamine	0.44	0.050	0.54	0.010	0.56	0.010	0.56	0.010	0.54	0.010
1-stearoyl-GPE 180	0.42	0.060	0.57	0.005	0.44	0.070	0.63	0.002	0.52	0.010
2-aminobutyrate	0.41	0.070	0.48	0.020	0.53	0.020	0.61	0.003	0.52	0.010
orotate	0.46	0.040	0.53	0.010	0.54	0.020	0.54	0.010	0.52	0.010
dihydroferulic acid	0.63	0.003	0.55	0.010	0.60	0.010	0.74	<0.001	0.50	0.010
phosphocholine	0.43	0.050	0.57	0.005	0.62	0.005	0.55	0.010	0.48	0.020
naringenin	0.54	0.010	0.45	0.030	0.49	0.030	0.57	0.010	0.47	0.020
cadaverine	0.50	0.020	0.56	0.010	0.52	0.020	0.50	0.020	0.46	0.030
ferulate	0.71	0.001	0.58	0.003	0.63	0.004	0.62	0.002	0.45	0.030
imidazole propionate	0.50	0.020	0.44	0.040	0.48	0.040	0.62	0.003	0.45	0.030
syringic acid	0.49	0.030	0.49	0.020	0.51	0.030	0.59	0.004	0.41	0.050
1-linoleoyl-GPE 182	0.49	0.030	0.45	0.040	0.52	0.030	0.54	0.010	0.37	0.090
2,4,6-trihydroxybenzoate	0.37	0.100	0.42	0.050	0.53	0.020	0.41	0.070	0.36	0.100
2-hydroxystearate	-0.51	0.020	-0.67	0.001	-0.74	<0.001	-0.39	0.080	-0.46	0.020
adenosine	-0.46	0.040	-0.55	0.010	-0.55	0.020	-0.38	0.100	-0.48	0.020

2-hydroxypalmitate	-0.50	0.020	-0.71	<0.001	-0.73	0.001	-0.39	0.080	-0.49	0.020
2'-deoxyguanosine	-0.46	0.040	-0.49	0.020	-0.50	0.030	-0.44	0.050	-0.49	0.020
retinol (vitamin A)	-0.42	0.060	-0.67	0.001	-0.54	0.020	-0.42	0.060	-0.52	0.010
N-butyroyl-sphingosine	-0.47	0.030	-0.69	<0.001	-0.67	0.002	-0.41	0.070	-0.54	0.010
4-methylthio-2-oxobutanoate	-0.61	0.003	-0.60	0.003	-0.49	0.030	-0.44	0.050	-0.54	0.010
N-palmitoyl-sphinganine d180/160	-0.57	0.010	-0.54	0.010	-0.40	0.100	-0.52	0.020	-0.57	0.004
N-palmitoyl-sphingosine d181/160	-0.56	0.010	-0.62	0.002	-0.50	0.030	-0.47	0.030	-0.58	0.003
N-methylalanine	-0.62	0.003	-0.66	0.001	-0.43	0.070	-0.49	0.020	-0.58	0.003
deoxycarnitine	-0.50	0.020	-0.59	0.003	-0.40	0.100	-0.53	0.010	-0.58	0.003
phytosphingosine	-0.49	0.030	-0.71	<0.001	-0.67	0.002	-0.42	0.070	-0.59	0.003
sphinganine	-0.50	0.020	-0.72	<0.001	-0.67	0.002	-0.42	0.060	-0.59	0.003
5,6-DiHETrE	-0.53	0.020	-0.71	<0.001	-0.57	0.010	-0.48	0.030	-0.61	0.002
tetradecanedioate C14	-0.62	0.003	-0.65	0.001	-0.56	0.010	-0.61	0.003	-0.62	0.002
stearoyl ethanolamide	-0.47	0.030	-0.60	0.002	-0.40	0.100	-0.40	0.080	-0.64	0.001
sphingosine	-0.57	0.010	-0.76	<0.001	-0.68	0.002	-0.48	0.030	-0.65	0.001
urate	-0.65	0.002	-0.73	<0.001	-0.59	0.010	-0.59	0.004	-0.67	<0.001
cholesterol	-0.64	0.003	-0.62	0.002	-0.63	0.005	-0.64	0.002	-0.67	<0.001
7-hydroxycholesterol alpha or beta	-0.56	0.010	-0.69	<0.001	-0.58	0.010	-0.64	0.002	-0.69	<0.001
dehydrolithocholate	-0.38	0.090	-0.61	0.002	-0.41	0.090	-0.43	0.060	-0.70	<0.001
indole	-0.50	0.020	-0.62	0.002	-0.43	0.080	-0.41	0.070	-0.71	<0.001
N-alpha-acetylornithine	-0.65	0.002	-0.79	<0.001	-0.68	0.002	-0.64	0.002	-0.73	<0.001
beta-alanine	-0.55	0.010	-0.59	0.003	-0.42	0.090	-0.63	0.002	-0.77	<0.001
